# Reproductive parasitism by worker honey bees suppressed by queens through regulation of worker mandibular secretions

**DOI:** 10.1038/s41598-018-26060-w

**Published:** 2018-05-24

**Authors:** Fiona N. Mumoki, Christian W. W. Pirk, Abdullahi A. Yusuf, Robin M. Crewe

**Affiliations:** Social Insects Research Group, Department of Zoology and Entomology, University of Pretoria, Private Bag X20, Hatfield, 0028 Pretoria, South Africa

## Abstract

Social cohesion in social insect colonies can be achieved through the use of chemical signals whose production is caste-specific and regulated by social contexts. In honey bees, queen mandibular gland pheromones (QMP) maintain reproductive dominance by inhibiting ovary activation and production of queen-like mandibular gland signals in workers. We investigated whether honey bee queens can control reproductively active workers of the intraspecific social parasite *Apis mellifera capensis*, parasitising *A. m. scutellata* host colonies. Our results show that the queen’s QMP suppresses ovarian activation and inhibits the production of QMP pheromone signals by the parasitic workers, achieved through differential expression of enzymes involved in the biosynthesis of these pheromones at two points in the biosynthetic pathway. This is the first report showing that honey bee queens can regulate reproduction in intraspecific social parasites and deepens our understanding of the molecular mechanisms involved in the regulation of worker reproduction in social insects.

## Introduction

Division of labour is one of the key characteristics of social insects, where the reproductive role is carried out by the queen who mates with males and lays the overwhelming majority of eggs, while workers carry out the routine tasks in the colony. This strict colony organisation, of which honey bees are an example, is maintained by use of chemical communication, with caste-related tasks and reproductive development mostly controlled by means of pheromones produced by the queen and brood^1,2^. While the reproductive role of the queen in most honey bee colonies is firmly maintained, especially in European subspecies of the Western honey bee (*Apis mellifera* L.), it is more complex in others, such as the African subspecies where the majority of the colonies are not kept in apiaries^3^. Workers are, in principle, able to activate their ovaries and lay unfertilised eggs that normally become drones^4^. The contribution of worker reproduction is rather small in the European honey bee^5^ but quite significant in African honeybees^6^, where many colony members evade the queen-induced sterility, activate their ovaries and lay unfertilised eggs that become drones^4^.

At the extreme end of this evasion is the Cape honey bee (*Apis mellifera capensis* Escholtz 1822). Workers of this subspecies have evolved an exceptional ability to give rise to female offspring through the process of thelytokous parthenogenesis^7–9^, a trait shown to be under the control of a single recessive locus (*thelytoky*, *th*) which segregates in a Mendelian fashion^10,11^. This locus (*th*) has also been shown to influence the development of certain queen-like phenotypes in the *A. m. capensis* laying workers including rapid ovary activation and queen pheromone synthesis^12–15^, enabling these (morphologically) worker honey bees to develop into false queens. Through a short-sighted evolutionary selection process^16^, a specific invasive lineage of *A. m. capensis* workers has developed into a facultative reproductive parasite^17–20^ of *A. m. scutellata* (Lepeletier 1836) colonies^7,21–23^. As facultative parasites, *A. m. capensis* workers do not reproduce while in the presence of their own queen^21^, but they actively seek out and gain entry into susceptible host colonies (e.g., queenless hosts)^24^, where they produce a queen-like pheromonal bouquet^25–28^ and activate their ovaries (therefore establishing themselves as false queens)^29–31^. In host colonies of honey bee subspecies other than *capensis*, a pheromonal contest between the host queen and the false queens ensues, usually leading to the death of the queen^32^. The clonal workers (=clones) then proceed to take over the reproductive role in the colony, leading to its eventual collapse. This is what has come to be known as the ‘Capensis problem’^17,18,27,33–35^. While we know that some host queens survive clone infestation, the specific conditions that favour host queen survival are still under investigation. In this work, we examine the effect that the presence of a host queen has on the development of dominance in the infesting *A. m. capensis* workers.

The queen’s pheromones are produced in various glands, with the mandibular glands producing one of the best described insect pheromone complexes; the queen mandibular gland pheromone (QMP) complex. QMP is part of a nine-compound pheromone blend that attracts workers to attend the queen^36–39^ and is composed of: methyl *p*-hydroxybenzoate (HOB), 9-oxo-2 (E)-decenoic acid (9-ODA), 4-hydroxy-3-methoxyphenylethanol (HVA), (*R,E*)-9-hydroxy-2-decenoic acid (9-HDA), (*S,E*)-9-hydroxy-2-decenoic acid (9-HDA), 10-hydroxy-2 (E)-decenoic acid and 10-hydroxydecanoic acid (10-HDAA)^39,40^. Functions of the QMP in the social organisation of the colony include: inhibiting worker ovary activation^41,42^, preventing rearing of new queens^38,43^, influencing age-related division of labour in worker bees by delaying honey bee behavioural maturation^44^, and controlling development of various physiological systems of young bees^45^. These functions have been well reviewed^37,40,46^.

**Figure 1:**
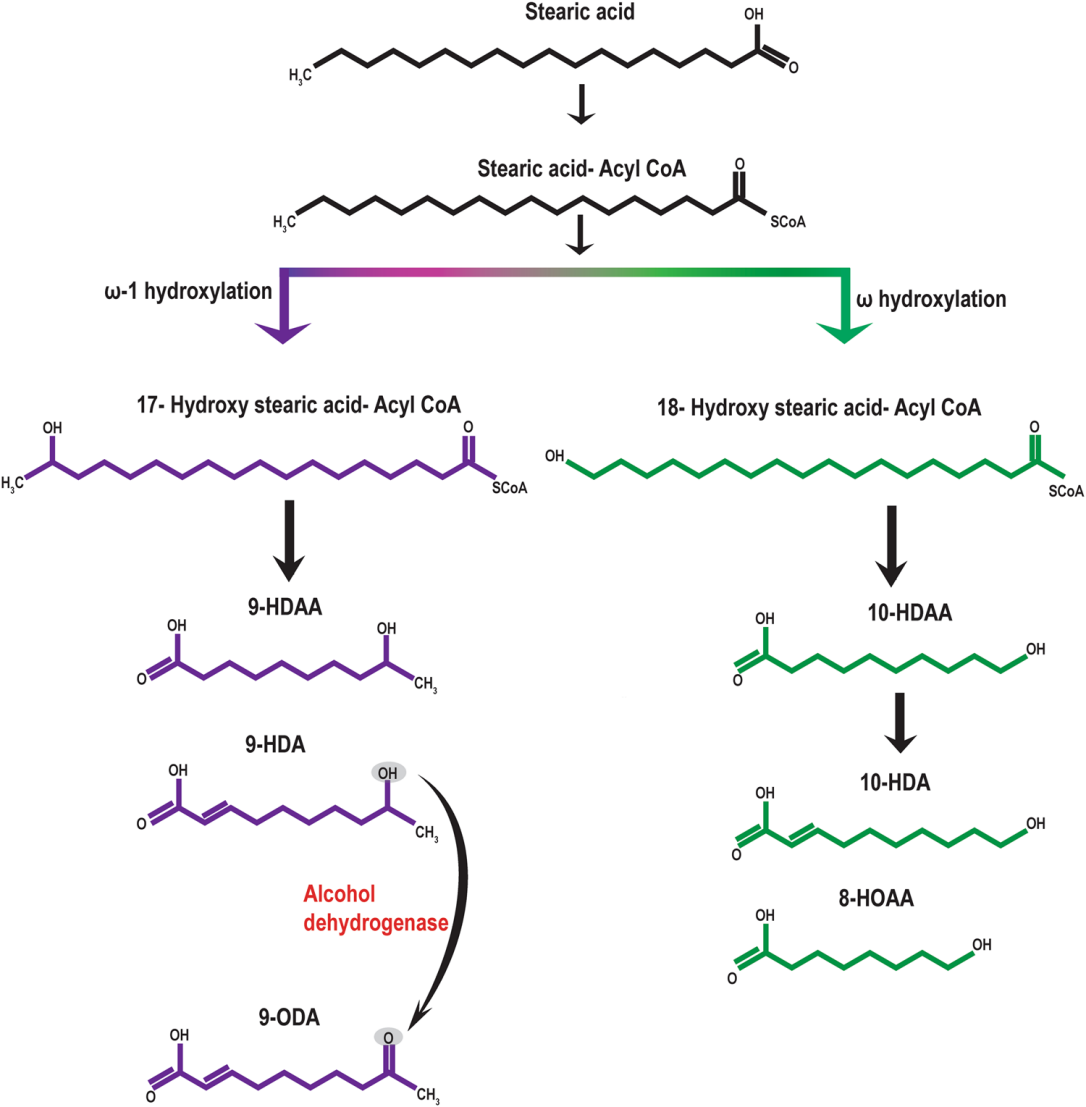
A schematic representation of the biosynthetic pathway of the main mandibular gland fatty acids in honey bee queens (ω-1 hydroxylation; purple) and queen right -workers (ω hydroxylation; green). The oxidative reduction of 9-HDA to 9-ODA is catalysed by the enzyme alcohol dehydrogenase.

Biosynthesis of the main components of the mandibular gland pheromones takes place in a caste-specific manner where stearic acid (octadecanoic acid), the principal starting material for queen substance, is converted into the main mandibular gland pheromone component through a bifurcated three-step process^47^ (Fig. 1). The first step is the hydroxylation (functionalisation) of stearic acid in either the ω or ω-1 positions (caste biased), followed by the chain shortening of the 18- and 17-hydroxystearic acids through β-oxidation. The final step is the oxidation of the ω and the ω-1 hydroxy group to give diacids and keto acids. The mandibular gland pheromones of honey bee workers have high amounts of 10-HDA and its precursor 10-HDAA, followed by HOB, 9-ODA and 9-HDA^48,49^. In contrast, queen mandibular gland pheromones have high amounts of 9-ODA, R and S 9-HDA, HOB and HVA, in this order, with 9-ODA being the predominant component^50^ (Fig. 1). This caste specific bifurcated pathway is achieved by the differential expression of various genes thought to participate in the biosynthesis of honey bee mandibular gland pheromones^46,51,52^.

There is great plasticity in the production and expression of pheromones in various groups of social insects^37,46,53^. The variations are based on social environment; presence of brood, presence or absence of the queen^1,54,55^ and physiological development; age^25^ and mating^50^. In honey bees for instance, the presence or absence of the queen (queenright or queenless colonies, respectively) has been shown to greatly affect pheromone expression in the worker bees from these hives (workers from queenright colonies = QR workers and those from queenless colonies = QL workers)^1,54,55^. The mandibular glands of typical queenright *A. mellifera* workers tend to have high amounts of 10-HDA and 10-HDAA^27,39,48,56^. In the absence of the queen, their queenless counterparts employ the molecular pathways ordinarily utilised by queens in production of 9-ODA through the conversion of its precursor 9-HDA into 9-ODA^13,51,52^. This reductive-oxidation of 9-HDA to 9-ODA is catalysed by the enzyme alcohol dehydrogenase (ADH)^52,57^. This group of enzymes (Alcohol dehydrogenases) are ubiquitous to almost all groups of living organisms, from Archaea^58^, yeast and bacteria^59^ to almost all eukaryotes^60^. ADHs perform a wide range of functions including biosynthesis and degradation of beneficial insect metabolites such as pheromones^61,62^ to the breakdown of toxins such as the oxidative metabolism of ethanol in the mammalian liver^63^. *Adh* transcripts have been identified in the honey bee mandibular glands where the enzyme is thought to catalyse pheromone biosynthetic reactions^52,57^ in the gut microbiome where it participates in fermentation^64^ and fat bodies where it participates in various cellular metabolism processes^65^. In the mandibular glands, the expression of ADH has been shown to be higher in queens as compared to workers from both queenright and queenless colonies^52,57^. Production of pheromone components typically found in queen mandibular glands allows workers to become egg-layers and regulate the reproductive capacity of their nest mates.

Here, we examined the effect that social environment has on the expression of reproductive dominance in *A. m. capensis* parasitic workers by recording the pheromone profiles and ovarian activation of field-collected parasitic workers obtained from queenright and queenless colonies. Further, we examined the levels of transcription of ADH, the enzyme responsible for the reductive-oxidation of 9-HDA to the ‘queen substance’ 9-ODA. Bearing in mind the high colony loses experienced annually by South African beekeepers due to the Capensis problem^22^, we hypothesised that, the development of reproductive parasitism by the *A. m. capensis* parasitic workers would be independent of the social environment of the host colonies, specifically presence or absence of *A. m. scutellata* queens. We predicted that that all parasitic workers, regardless of the social environment of the host colony, would produce queen-like mandibular gland pheromones.

## Results

### Ovarian activation status and presence of spermatheca

All (100%) *A. m. capensis* laying workers collected from both queenright (QR) and queenless (QL) colonies showed the presence of a spermatheca.

**Figure 2:**
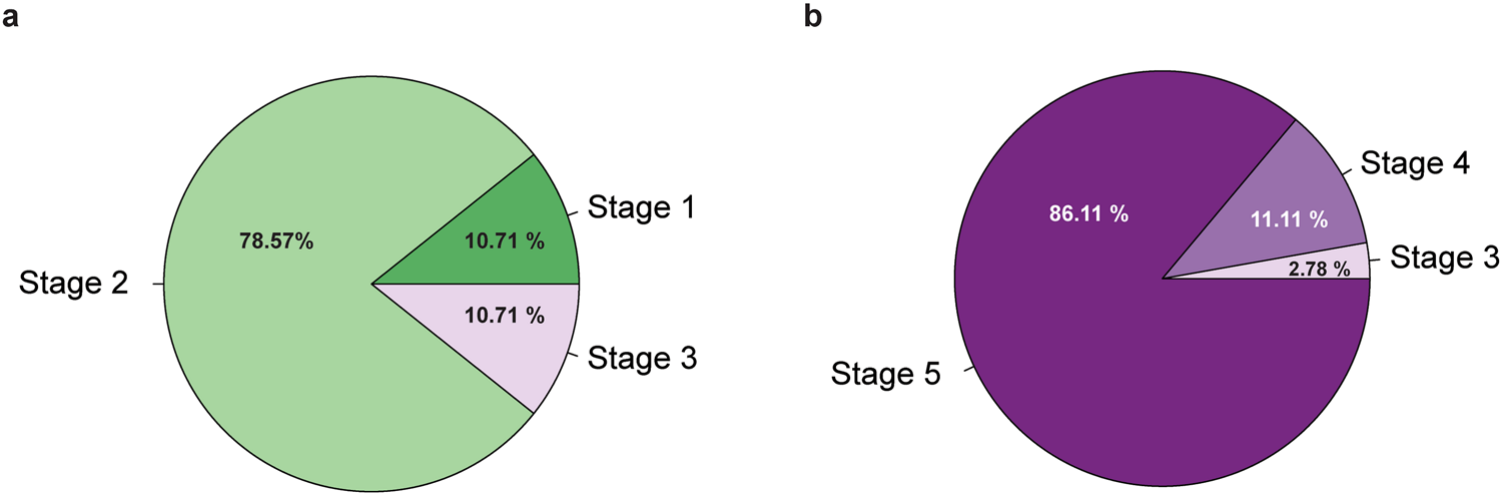
Ovary activation status for field-collected *Apis mellifera capensis* parasitic workers from queen-right (QR) (**a**) and queen-less (QL) (**b**). *A. m. scutellata* colonies. Stage I & II = threadlike ovarioles, III = intermediate with early oocyte development, IV & V = clearly developed oocytes.

Ovarian activation differed (MWU, U = 1.50, N_QR_ = 28, N_QL_ = 36, p < 0.0001) between clones collected from the QR and QL colonies (Fig. 2). Clones from QR colonies only had stage I, II and III ovaries (Fig. 2a). As only stage III, IV and V are considered to be activated ovaries, only 10.71% of the QR clones had activated ovaries. In contrast, clones collected from QL colonies displayed stage III, IV and V ovaries (Fig. 2b). Therefore, all the clones from QL colonies had activated ovaries with 86.11% having fully developed oocytes in their ovarioles.

### Variation in pheromone profiles of *A. m. capensis* parasitic workers from QL and QR *A. m. scutellata colonies*

**Figure 3:**
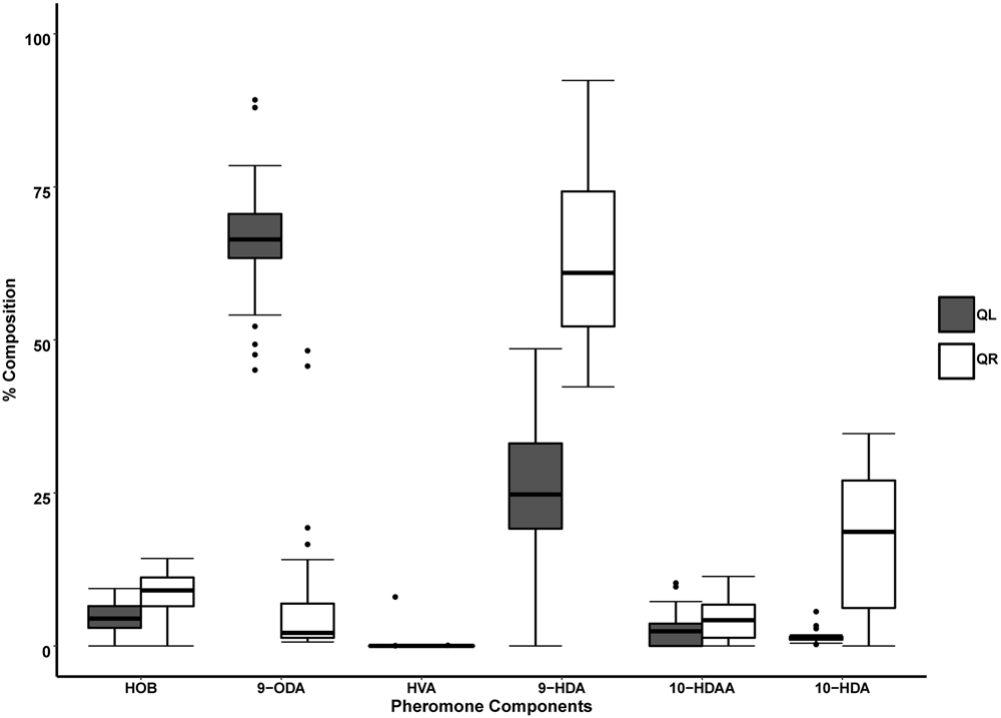
Percentage composition of the six mandibular gland components (HOB, 9-ODA, HVA, 9-HDA, 10-HDAA and 10-HDA) of field-collected *Apis mellifera capensis* clones from queenless (closed bars) and queenright (open bars) *A*. *m. scutellata* colonies (▬ = mean, ▯ = 25–75%, ⌶ = min-max, ● = outliers).

Mandibular gland pheromone profiles of clones from queen-right (QR) and queen-less (QL) colonies of *A. m. scutellata* differed significantly. In QL clones, the queen substance 9-ODA was the most abundant fatty acid component making up 66.18 ± 1.64% of the mandibular gland contents, followed by its precursor 9-HDA (25.46 ± 1.77%). This was in contrast to the profile of QR clones where the most abundant components were 9-HDA (63.64 ± 3.00%), 10-HDA (15.34 ± 2.11%) and 9-ODA (8.08 ± 2.26%), respectively (Fig. 3).

**Figure 4:**
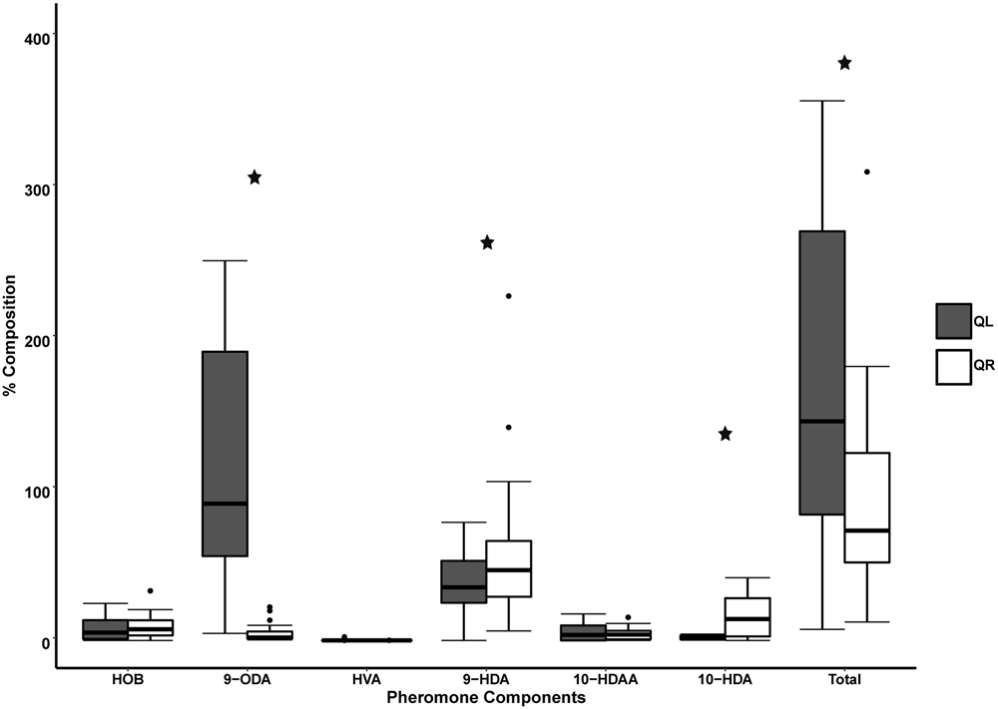
Amounts of six mandibular gland components (HOB, 9-ODA, HVA, 9-HDA, 10-HDAA and 10-HDA) of field-collected *Apis mellifera capensis* clones from queenless (closed bars) and queenright (open bars) *A. m. scutellata* colonies (▬ = mean, ▯ = 25–75%, ⌶ = min-max, ● = outliers, *p ≤ 0.05 indicates statistical significance).

Comparative analyses of the quantities of the six different components in the QR and QL social condition revealed that there were significant differences in the expression patterns of the pheromones given different social conditions (Fig. 4). The mean total amount of fatty acids produced were significantly higher in QL clones (163.53 ± 17.71 μg) as compared to QR clones (91.71 ± 11.89 µg), (Mann-Whitney U Test (w/continuity correction; Z-adjusted = −2.686 N_QR_ = 28; N_QL_ = 36, p = 0.006). The mean amount of 9-ODA was significantly higher (MWU; Z-adjusted = −6.38, N_QR_ = 28, N_QL_ = 36, p < 0.0001) in the QL clones than in the QR clones, while both the amounts of 9-HDA (MWU: U = 358.00, Z-adjusted = 1.969, N_QR_ = 28, N_QL_ = 36, p = 0.048) and 10-HDA (MWU: Z-adjusted = 3.98, N_QR_ = 28, N_QL_ = 36, p = 0.0004) were significantly lower in the QL Clones as compared to QR clones. There was no significant difference in the mean amounts of HOB (MWU: Z-adjusted = 3.98, N_QR_ = 28, N_QL_ = 36, p = 0.3853), HVA (MWU: Z-adjusted = −0.311, N_QR_ = 28, N_QL_ = 36, p = 0.7521) and 10-HDAA (MWU: Z-adjusted = 0.237, N_QR_ = 28, N_QL_ = 36, p = 0.8141) in workers from the two different social conditions.

Regardless of whether the clones were from QR or QL colonies, there were significant differences between individuals with activated (AO) and inactivated ovaries (IO), for the expression of 9-ODA (MWU, U = 60.5, N_AO_ = 25, N_IO_ = 39 P < 0.0001), 9-HDA (MWU, U = 282, N_AO_ = 25, N_IO_ = 39, P = 0.004) and 10-HDA (MWU, U = 135, N_AO_ = 25, N_IO_ = 39, p < 0.0001) but no significant differences in the expression of HOB, HVA, 10-HDAA or the total pheromone amounts for these honey bees.

### Classification of pheromone profiles into queen or worker signals

**Figure 5:**
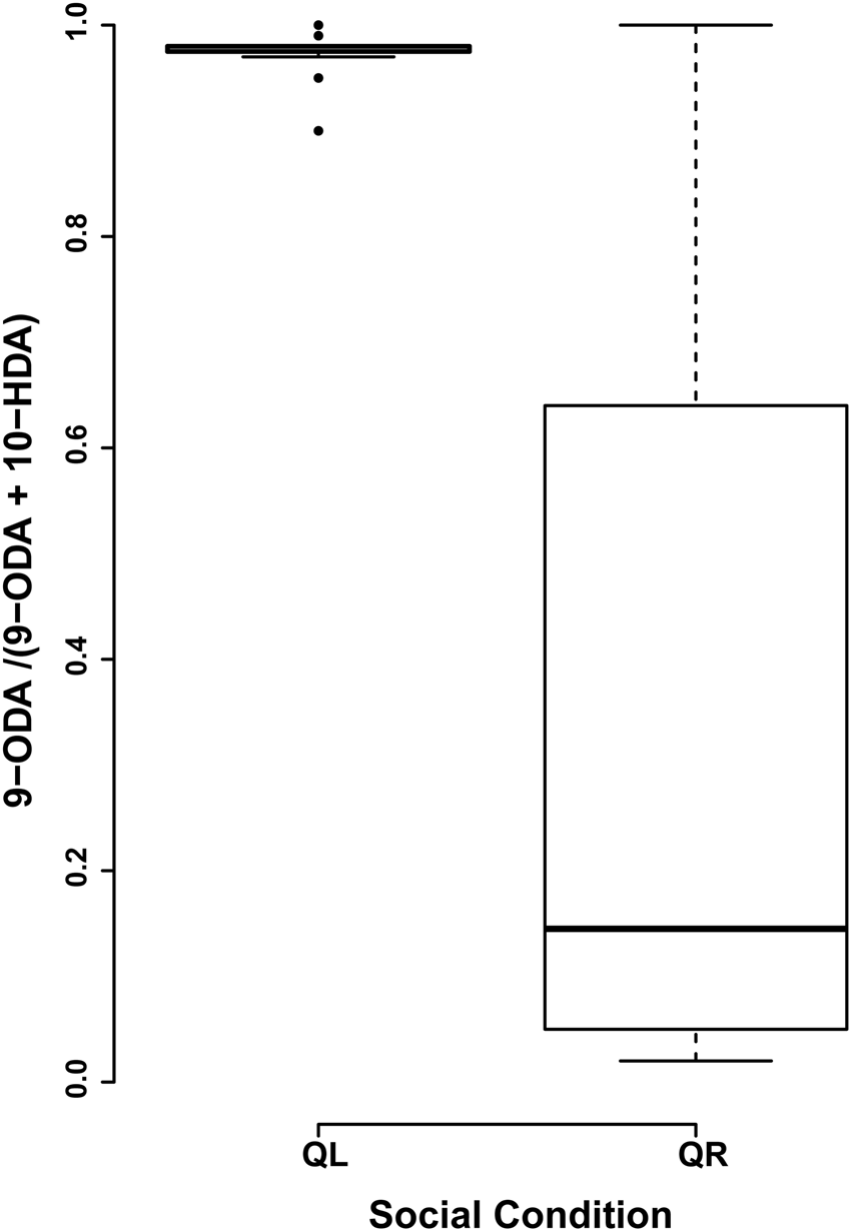
The pheromone component ratios in parasitic *A. m. capensis* workers collected from queen-less (QL) and queen-right (QR) colonies of *A.m. scutellata*. Error bars are SD from the means. A queen-like signal has a ratio of >0.8–1, and a worker-like signal has a ratio of >0.5.

Pheromone profiles produced by individuals were classified based on the ratio of the major components. The ratio 9-ODA/(9-ODA + 10-HDA) revealed that pheromone profiles of the QR parasitic *A. m. capensis* workers were mostly worker-like (0.17 ± 0.23) while their QL counterparts (0.98 ± 0.02) had only queen-like profiles (Fig. 5). The variation in pheromone ratios was significantly different (MWU, U = 44.00, N_QR_ = 28, N_QL_ = 36, p < 0.0001).

### Expression of alcohol dehydrogenase (ADH) transcripts

**Figure 6:**
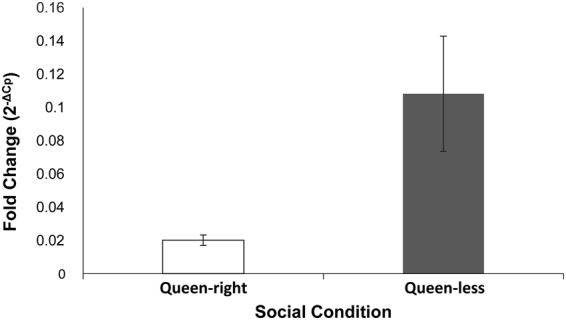
Change in levels of gene expression of the alcohol dehydrogenase (normalized to the reference gene *Cyp4g11*) in queen-right (QR; open bar) and queen-less (QL; closed bar) clones. Error bars represent standard error of fold change (2^−ΔCp^).

There were approximately five times as many transcripts of alcohol dehydrogenase in QL *A. m. capensis* parasitic workers as compared to those of the QR workers, showing that the higher levels of alcohol dehydrogenase in dominant workers occurred in the absence of the influence of regulatory pheromones from a queen. There was no significant difference in the *Cyp4g11* (endogenous control) in either QL or QR workers (Fig. 6).

## Discussion

The *A. m. capensis* laying workers that act as social parasites, gain entry into host colonies, produce queen-like multi-glandular pheromone secretions^66^ and take over the role of reproduction from host queens^29–31^, eventually leading to the collapse of infested colonies. Here, we show that the presence or absence of a queen in the infested colonies plays a critical role in determining whether the *A. m. capensis* workers (which are already predisposed to social parasitism) develop into fully fledged reproductive parasites. The regulation of pheromonal biosynthetic pathways in these workers by the queen pheromone is shown to regulate worker reproduction in these honey bee colonies.

The presence of a spermatheca (a queen-associated trait) was confirmed in all the *A. m. capensis* parasitic workers from both the QR and QL colonies, although visually it was noted that the size of the spermatheca differed in different individuals. While spermathecae were not measured for this study, previous studies recorded the size of the spermatheca in *A. m. capensis* workers to range between 0.37 mm and 0.55 mm^67^. As these were field-collected bees of varying ages, the differences in the size of the spermatheca may be as a result of age or nutritional/feeding differences experienced by the *A. m. capensis* parasites during their developmental stages^68^ and varied geographically^67^. The spermatheca has been used before as a key trait in the identification of the parasitic laying *A. m. capensis* worker^8,25,67^.

Ovarian activation clearly differed in the QL and QR clones. While all the QL clones had activated ovaries; stage III, IV and V (with majority of the clones having stage V), only 10% of QR colonies had activated ovaries (at stage III), showing that the presence of the *A. m. scutellata* queen indeed influenced the reproductive condition of the *capensis* clones. Various factors influence ovarian activation in honey bees, including environmental factors such as nutrition^69^ and the individual’s physiological status such as age^70^. The greatest influence on suppression of ovarian activation is the influence of pheromones from the queen and brood^41,42,71^. The queen employs pheromones from various organs to suppress worker reproduction^41,66,72^, with the mandibular gland pheromones playing a crucial role as a ‘suppressive agent’ effectively inhibiting worker reproduction^73^.

The role of QMP in the inhibition of ovarian activation has been well documented^41,42^, for queens inhibiting ovarian activation within their own colonies. QMP components 9-ODA and 9-HDA have been shown to inhibit ovarian activation in both *Apis mellifera* and *Apis cerana*^74^. Other glands contributing to the queen’s overall pheromone bouquet such as the tergal glands^25,66,72^ also contribute to the suppression of reproductive dominance in workers through inhibition of both ovarian activation and production of queen-like pheromone signals. The components 9-ODA, 9-HDA and 10-HDA were seen to vary significantly with ovarian activation. These pheromone signals have been seen to covary with ovarian activation in *A. m. capensis*^31,69^ and are indeed associated with reproductive dominance. Schafer *et al*. in 2006 showed that pheromone dominance influenced the food consumed by (dominant) *A. m. capensis* workers who were fed through trophallaxis by the host workers. It is this protein-rich pre-processed ‘jelly-like’ pollen meal that enables the reproductively dominant *A. m. capensis* workers to dedicate most of their energies to activating their ovaries and laying eggs^69,75^. A component of honey bee brood pheromones, (E)-beta ocimene, has also been shown to play a role in suppression of ovarian activation in honey bee workers^76^. The effect that the presence of brood may have played in suppression of ovarian activation in these field-collected parasitic workers was not examined in this study but since there was brood in both sets of colonies, we expect that brood on its own did not inhibit ovarian activation of the false queens in the queen right colonies. This premise is further supported by studies on caged *A. m. scutellata* which showed that exposure to brood pheromone had no effect on ovarian activation in the caged bees^77^.

The pheromone profiles of the QR and QL parasitic *A. m. capensis* workers revealed that the presence of the queen had a significant influence on the pheromone profile of the parasites. Pheromone profiles of the QL *capensis* laying workers were dominated by fatty acid components typical of queens, such as 9-ODA and 9-HDA while those of QR clones were dominated by 9-HDA and 10-HDA. This QR (worker-like) profile has been described before by^27^ when assessing QR *A. m. capensis* (non-laying) workers under the control of an *A. m. capensis* queen, in the Cape region (endemic region of the *A. m. capensis* subspecies). The profiles produced by the QR Clones reported here are more similar to those produced by the QR *A. m. capensis* (non-laying) workers from the Stellenbosch region as reported by Zheng *et al*.^27^. This clearly demonstrates that *A. m. scutellata* queens have the ability to control reproductive dominance in Capensis parasites to the same extent that *A. m. capensis* queens control reproduction in non-laying Capensis workers. Since capensis social parasites can takeover colonies of other subspecies (reviewed in^30^ and^78^), the ability of the host queen to exercise a level of control suggests that other factors play a role in determining whether social parasites are able to take over a colony. These factors may include stress factors (such as pests and diseases), nutrition, various physiological states of the queen, colony structure (ratio of older to younger bees) and the amount and age of brood. Further, evidence shows that the social environment from which drifting (and host-seeking) *A. m. capensis* workers come, may have an influence on the ability of these workers to develop into reproductive individuals. *Capensis* workers drifting from queenless colonies were better able to develop into reproductives, with queen-like pheromonal ratios and activated ovaries^79^. This suggests that as *capensis* workers from queenright colonies were already under the influence of the queen from their own original colonies (possibly with near worker-like pheromone ratios and inactive ovaries) it was much easier for host queens in the infested host colonies to control these drifting parasitic workers.

The fatty acid 10-HDA was produced in larger quantities in the QR clones as compared to clones from QL colonies (Fig. 4). Although produced by both queens and workers, this fatty acid is produced in the highest proportions in worker mandibular glands^47^ and is a key constituent of royal jelly^80^. The ratio of 9-ODA:10-HDA for queenright *A. m. capensis* workers has been found to range between 0.3 and 0.1^27,79^. Only 67% (n = 19) of our QR parasitic clones fell within this range (0.17 ± 0.23). A second ratio (9-ODA/9-ODA + 10-HDA) widely used to assess the “queen-likeness” of honey bee mandibular gland signals^18,69^, also showed that only 75% (n = 21) of the QR worker signals were “worker-like”. This means that the rest of the QR workers and (100%) of the QL workers had activated the ‘queen-specific’ biosynthetic pathway, enabling them to produce higher quantities of the queen pheromone 9-ODA relative to the worker-like component 10-HDA^47^.

As expected, the proportion of 9-ODA produced was significantly higher in QL clones than in their QR counterparts as the queen is not present to suppress production of ‘queen substance’ in the QL colonies^32^. However, the amount of the 9-HDA, which is the precursor compound to 9-ODA was significantly higher in the QR workers as compared to the QL bees. This suggests that the queen’s influence on fatty acid biosynthesis in the clone workers’ mandibular glands is exercised at the level of halting the oxidation of 9-HDA into 9-ODA, rather than being able to prevent the clones from using the ω-1 hydroxylation route in the biosynthesis of mandibular gland pheromones (this is the pathway normally upregulated in queens and reproductively dominant workers)^51,52^. Queens prevent the formation of 9-ODA by inhibiting the production of the enzyme alcohol dehydrogenase^52,57^. The production of this enzyme was significantly higher in QL clones than in QR clones, indicating inhibition or suppression of the production of 9-ODA, by the *A. m. scutellata* queen. As the two arms of the bifurcated biosynthetic pathway are independent of each other^47^, the higher amounts of 9-ODA in QL clones as opposed to the QR clones could only come from the enzymatic oxidative-reduction of 9-HDA to 9-ODA a reaction that is inhibited in the worker caste. The low amounts of alcohol dehydrogenase in QR workers suggests that one mechanism employed by the queen to regulate the production of 9-ODA is suppression of the synthesis of this enzyme, resulting in an accumulation of the precursor 9-HDA. Alcohol dehydrogenase has indeed been shown to be highly expressed in the mandibular glands of queens as opposed to those of workers^52,57^. As the amount of 9-ODA has been shown to increase with age in both virgin and mated queens^50^, it follows that the amount of alcohol dehydrogenase converting 9-HDA to 9-ODA would increase with the age of the queens as well. Due to the fact that we measured the transcripts of ADH in field collected samples of workers whose ages were undetermined, we cannot rule out the possible effect that age of the *A. m. capensis* parasites had on the expression of ADH. Further, alcohol dehydrogenase has been shown to participate in various other key functions in insect communication, such as pheromone degradation as in the antennae of the moth *Maduca sexta*^62,81^. Even though our analyses were highly targeted to the honey bee mandibular glands, we cannot rule out the effect that other key metabolic functions may have on the expression of this enzyme.

All these pieces of evidence are indicative of intraspecific control of reproductive dominance where the *A. m. scutellata* queen controls dominance by inhibiting both ovarian activation and the production of queen-like pheromone signals. This is the first report documenting intraspecific control of reproductive dominance between subspecies through suppression of ovarian activation, as we have shown that the *Apis mellifera scutellata* queen was able to prevent ovarian activation in *Apis mellifera capensis* workers. Our work provides an insight into the pheromonal interplay between invasive Capensis social parasites and the queens of host colonies in their quest for reproductive dominance. We have shown that the presence of host queens of *Apis mellifera scutellata* inhibits both ovarian activation and oxidation of 9-HDA to 9-ODA in parasitic clone workers. In queenright colonies of most honey bee subspecies, the QMP of the queen prevents the workers from expressing the part of the biosynthetic pathway (Fig. 1) that results in the ω-1 hydroxylation of stearic acid. If this inhibition fails, then it appears from our work that the QMP can still inhibit the production of the ‘queen substance’ in these workers through blocking the production of alcohol dehydrogenase that would result in the conversion of 9HDA to 9ODA, thus revealing an ongoing battle for reproductive dominance that takes place in clone-infested colonies.

There is evidence from laboratory based experiments showing that *A. m. capensis* queens are better able to regulate production of dominance signals from the invasive clones than *A. m. scutellata* queens are^18^, possibly owing to the higher amounts of ‘queen substance’ produced by the Capensis queens^82^. The fact that South African beekeepers lose more *A. m. scutellata* colonies than *A. m. capensis* colonies due to the ‘Capensis problem’ regardless of colony management practices^22^, suggests that the results from the laboratory experiments are reflected in the field. Our results show that given the right colony conditions the *A. m. scutellata* queens can prevent a clone reproductive takeover. The right colony conditions could include sufficient amounts of host colony brood as brood pheromones potentially act as ‘honest signals’ or queen fertility indicators^73,83^, in addition to suppression of ovarian activation achieved by having the queen present in the colony^76^.

This work demonstrates that reproduction by invasive parasitic lineages is facilitated by the absence of the queen, and that in the presence of the queen there is a pheromonal contest between the queen and the parasitic workers that is dependent on the queen remaining in place. On queen loss, the parasitic workers are able to monopolise reproduction in the colony at the expense of the queen’s offspring.

## Materials and Methods

### Experimental Procedures

#### Honey bee samples

Colonies of *A. m. scutellata* honey bees infested by *A. m. capensis* clones were donated by local beekeepers from Gauteng and Limpopo provinces of South Africa. These were kept in quarantine in a restriction tent to stop them from flying freely and potentially infesting other colonies. They were then managed throughout the experimental period using standard beekeeping procedures^84^. Colonies were inspected for the presence or absence of a queen; either by visually searching and locating the queen or by looking for recent queen-laid eggs in unsealed brood cells which signifies the presence of a queen. Adult *A. m. capensis* clone workers (=clones) (tentatively identified as black bees among the typical yellow-black *A. m. scutellata* workers) were aspirated into collection jars from the frames of the infested colonies. Clones collected from queenright *A. m. scutellata* colonies were referred to as QR clones while those from queenless colonies were termed QL clones. The collected clones were then frozen and the heads removed for both pheromone analysis using gas chromatography and gene expression studies, while the abdomens were dissected for assessment of ovary activation and presence or absence of spermatheca. Presence of the spermatheca (a queen-associated trait) was used as a distinguishing feature between the *A. m. capensis* clones and *A. m. scutellata* bees^8,25^. A total of 112 clones were examined in this study, 60 from *A. m. scutellata* colonies without queens and 52 from queen-right colonies. Of these, 48 individuals (24 from each group) were used for the gene expression analysis while a total of 64 individuals (28 QR and 36 QL) were used for the pheromone analysis.

#### Dissection and extraction of mandibular glands

The honey bees were immobilised by freezing at −20 °C. The heads were then removed and put on ice while the thorax and abdomen placed on wax plates containing Insect Ringer pH 7.4 (6.4 mL 5 M NaCL, 3.75 mL 0.1 M CaCL_2_, 1.25 mL 1 M KCL) to facilitate abdominal dissections. For gene expression analysis, mandibular gland dissection was done as described by^85^ and the removed glands placed in an Eppendorf Tube^®^ (Hamburg, Germany) containing 200 µL of TRIzol^®^ Reagent (Invitrogen, Carlsbad 92008, USA) and stored at −80 °C awaiting RNA isolation. For GC analyses heads were placed in a glass vial containing 200 µL of dichloromethane, DCM (HPLC grade, Sigma-Aldrich, Chemie GmbH München, Germany) and extracted for at least 24 hours.

#### Assessment of ovary activation and presence of spermatheca

Abdominal dissection was carried out using standard techniques to expose the ovaries and spermatheca^85^. Ovaries were classified into one of five stages as described by^86^; stage I & II having threadlike ovarioles, III being intermediate with early oocyte development, IV & V with clearly developed oocytes^25,27,69,87^. Presence of spermathecae (a queen-associated trait) was also recorded^67^.

#### Gas Chromatographic analysis of mandibular gland pheromones

Heads were extracted in 200 µL of DCM at −20 °C for at least 24 hours. Before the start of the chromatographic analysis, the 200 µL head extract was divided into two, with 100 µL stored as backup, should there be need for further confirmation or analyses. The other 100 µL was evaporated to dryness under a steady stream of charcoal-filtered nitrogen and GC analysis carried out using the procedures reported in^18,48,88^ with slight modifications as follows: the residues were re-dissolved in 10 µL of internal standard solution (~1 mg octanoic acid and ~1 mg tetradecane in 4 mL DCM). To this, 10 µL of bis-(trimethylsily) trifluoroacetamide, BSTFA (Sigma-Aldrich, Chemie Gmbh München, Germany) was added to derivatise the fatty acids. Separation of the mandibular gland pheromones was carried out using an Agilent 6890N Gas Chromatograph (GC), in the split-less mode on a methyl silicone coated fused silica column (HP-1MS, 25 m × 0.20 mm × 0.33 µm). Helium with a constant flow rate of 1 mL per minute was used as a carrier gas. The temperature of the oven was programmed at 60 °C for 1 min, then increased to 100 °C at 50 °C per min and to 220 °C at a rate of 3 °C minute. This final temperature was maintained for 10 minutes. Identification of the constituent pheromones was based on comparisons of the retention times of the analytes with those of known synthetic mandibular gland pheromone standards. Whilst quantification was achieved relative to the mass ratios of the internal standard mixture.

#### Classification of pheromone profiles into queen or worker pheromone signals

To assess how queen-like or worker-like mandibular gland profiles from the QL and QR clones were, ratios of the amount of the queen substance (9-ODA) to those of the worker component (10-HDA) were computed as follows: 9-ODA/(9-ODA + 10-HDA). Where a ratio of <0.5 was classified as worker-like, >0.5 ≤ 0.7 considered to be intermediate and >0.8–1.0 queen-like^18,69^

#### Expression of the alcohol dehydrogenase (Adh) gene

Primer synthesis: The honey bee alcohol dehydrogenase (*Adh*) coding sequence was obtained from GenBank (accession number GB15375) and primers flanking the coding region designed using Primer3 Plus Software (www.primer3plus.com) and by manual curation. The sequence of the primers designed were: F; 5′ GCT TCC TGC TGT AGG AAA TAG AGC 3′ and R; 5′ CTT GTT TCT CCA TTT CGG CCC 3′. The *Adh* mRNA is made up of 4 exons and the primer set designed here spans the end of exon 2 and extend to half of exon 3, amplifying a fragment that is 218 bp long. To test the designed primers, cDNA from *A. m. capensis* was used in PCR amplification using the gene-specific primers and the amplicons purified and then sequenced. The identity of the gene region amplified was confirmed through homology searching against the GenBank repository where it was subsequently deposited with the accession number MF144184.

*Cyp4g11* was used as the endogenous control as it was found to be constitutively expressed in the mandibular glands of queens and also of workers from queen-less and queen-right colonies^51^. The primer sequence for this fragment was: F; 5′ GGC TGT AAT GAA GAT GTG CGA C 3′ and R; 5′ GTG CGC TAT TAT CAA TGA TGT TAC G 3′.

#### RNA isolation and purification

RNA isolation from the mandibular glands was done as follows; pairs of glands were homogenised in 200 μL of TRIzol^®^ Reagent. Chloroform and Isoamyl Alcohol (Merck KGaA, Darmstadt, Germany) were used to achieve phase separation, after which the aqueous phase was removed and to it 180 µL of ice-cold isopropanol added to precipitate the RNA at −80 °C overnight. Washes were carried out using 85% molecular grade Ethanol (Merck KGaA Darmstadt, Germany) and RNA re-suspended in 40 µL of nuclease-free water.

Degradation of any co-precipitated DNA was carried out using the DNase I kit (Invitrogen, Carlsbad 92008, USA) following manufacturer’s instructions. The quality and quantity of the resultant DNA-free RNA was checked using a Nanodrop 2000 (Waltham Massachusetts, USA). Sets (n = 3; biological replicates) of mandibular gland RNA pooled from eight individuals were made and the RNA for cDNA synthesis normalised to 300 ng.

#### cDNA synthesis and qPCR

cDNA synthesis was carried out using the Superscript IV cDNA synthesis kit (Invitrogen, Carlsbad 92008, USA), using 18-mer oligo dT primers (Thermo Scientific). The following protocol was used; 1x SSIV Buffer, 2.5 μM Oligo dT, 0.5 mM dNTP mix, 5 mM dTT, 2 U/µL RNaseOUT™ Rnase Inhibitor, 2 U/µL of SSIV reverse transcriptase and water to top up to 20 µL. The thermocycler regimen consisted of 65 °C for 5 min (primer-template mix) then incubated on ice for 1 min after which the dNTPs, Oligo dT, Rnase Inhibitor and reverse transcriptase were added. The thermocycler program used for the cDNA synthesis was 23 °C for 10 min, 52 °C for 10 min, 80 °C for 10 min and 4 °C to hold.

Quantitative PCR was done using the PowerUP qPCR kit (Applied Biosystems, Foster City, California, USA) using the LightCycler^®^ 1.5 Instrument II Real Time PCR thermocycler (Roche, Basel Switzerland) in a 20 µL reaction volume with 1X PowerUP SYBR mix, 10 pmoles/µL of each primer, 3 µL of the cDNA template and water to top up to 20 µL. The thermocycler regimen used was as followed; 95 °C for 2 min for enzyme denaturation, 55 cycles of 95 °C for 15 seconds, and 60 °C for 30 seconds (fluorescence collected), followed by a standard dissociation program.

### Statistical and data analyses

Normality was tested using the Shapiro-Wilk test. Due to non-normal distribution of the honey bee mandibular gland profiles non-parametric tests were used for all downstream statistical analyses^89^.

Mann Whitney U test was used to assess the difference in ovary activation and expression of each of the constituents of the mandibular gland pheromones. Mann Whitney U was also used to carry out pairwise comparisons of the pheromone expression levels for each of the six components in both QR (in the presence of the queen) and QL (absence of the queen) social conditions, and to assess whether there are any overall significant differences in the pheromone ratios between QR and QL clones. Kruskal-Wallis test was carried out to assess the differences in total amounts for each of the MG pheromone components in QR and QL clones. Statistical significance was set at α < 0.05.

For the gene expression analyses, homogeneity in the amplification of the genes was analysed by examining the melt curves of the amplified genes. Standard curves were constructed by assessing the amplification trends of cDNA for the target and standard genes, covering 100-fold dilution concentrations. The mean normalised expression values of each target gene were calculated by comparing its threshold cycle (C_p_) against those of the reference genes, as described for the 2^−∆Cp^ method^90^, where ∆Cp = the C_p_ of *Adh* - C_p_ of *Cyp4g11* and fold change in the expression of *Adh* in clones from QL and QR *A. m. scutellata* colonies calculated as 2^−∆Cp^ (QL Clones)/2^−∆Cp^ (QR Clones). Differences in gene expression were inferred from non-overlapping standard error.

All statistical analyses were carried out in R Environment version 3.4.0^91^.

### Data availability

All the datasets generated and analysed in this study are available from the corresponding author upon reasonable request.

## References

[1] Winston, M. L. *The Biology of the Honey Bee*. (Harvard University Press, 1987).

[2] Pirk, C. W., Sole, C. L. & Crewe, R. *Honeybees of Asia in Pheromones* (eds Hepburn, H. R. & Radloff, S. E.) 207–214 (Springer, 2011).

[3] Pirk CWW, Crewe RM, Moritz RFA (2017). Risks and benefits of the biological interface between managed and wild bee pollinators. Functional Ecology.

[4] Crozier, R. H. Animal CytogeneticsVol. 3 in *Hymenoptera Ch*. Insecta, 17–36 (1975).

[5] Visscher PK (1996). Reproductive conflict in honey bees: a stalemate of worker egg-laying and policing. Behav Ecol Sociobiol.

[6] Moritz R, Beye M, Hepburn H (1998). Estimating the contribution of laying workers to population fitness in African honeybees (*Apis mellifera*) with molecular markers. Insect. Soc..

[7] Onions G (1912). South African ‘fertile worker bees’. South Afrrican Agriculture Journal.

[8] Hepburn HR, Crewe RM (1991). Portrait of the Cape honeybee, *Apis mellifera capensis*. Apidologie.

[9] Ruttner F (1977). The Problem of the Cape Bee (*Apis Mellifera Capensis* Escholtz): Parthenogenesis — Size Of Population — Evolution. Apidologie.

[10] Aumer D, Allsopp MH, Lattorff HMG, Moritz RFA, Jarosch-Perlow A (2017). Thelytoky in Cape honeybees (*Apis mellifera capensis*) is controlled by a single recessive locus. Apidologie.

[11] Lattorff H, Moritz R, Fuchs S (2005). A single locus determines thelytokous parthenogenesis of laying honeybee workers (*Apis mellifera capensis*). Heredity.

[12] Lattorff HMG, Moritz RFA, Crewe RM, Solignac M (2007). Control of reproductive dominance by the thelytoky gene in honeybees. Biology Letters.

[13] Jarosch A, Stolle E, Crewe RM, Moritz RFA (2011). Alternative splicing of a single transcription factor drives selfish reproductive behavior in honeybee workers (*Apis mellifera*). Proceedings of the National Academy of Sciences.

[14] Hemmling C, Koeniger N, Ruttner F (1979). Quantitative Bestimmung der 9-oxodecensäure im Lebenszyklus der Kapbiene (*Apis mellifera capensis* Escholtz). Apidologie.

[15] Ruttner F, Hesse B (1981). Rassenspezifische unterschiede in ovarentwicklung und eiablage von weisellosen arbeiterinnen der honigbiene *Apis mellifera* L. Apidologie.

[16] Moritz RF, Pirk CW, Hepburn HR, Neumann P (2008). Short-sighted evolution of virulence in parasitic honeybee workers (*Apis mellifera capensis* Esch.). Naturwissenschaften.

[17] Dietemann V, Neumann P, Härtel S, Pirk CWW, Crewe RM (2007). Pheromonal dominance and the selection of a socially parasitic honeybee worker lineage (*Apis mellifera capensis* Esch.). Journal of Evolutionary Biology.

[18] Dietemann V, Pflugfelder J, Härtel S, Neumann P, Crewe RM (2006). Social parasitism by honeybee workers (*Apis mellifera capensis* Esch.): evidence for pheromonal resistance to host queen’s signals. Behav Ecol Sociobiol.

[19] Hepburn H, Allsopp M (1994). Reproductive conflict between honeybees: Usurpation of *Apis mellifera scutellata* colonies by *Apis mellifera capensis*. South African Journal of Science.

[20] Lundie A (1954). Laying worker bees produce worker bees. South African Bee Journal.

[21] Härtel S, Neumann P, Raassen FS, Moritz RFA, Hepburn HR (2006). Social parasitism by Cape honeybee workers in colonies of their own subspecies (*Apis mellifera capensis* Esch.). Insect. Soc..

[22] Pirk CWW, Human H, Crewe RM (2014). & vanEngelsdorp, D. A survey of managed honey bee colony losses in the Republic of South Africa–2009 to 2011. Journal of Apicultural Research.

[23] Woyke, J. Invasion of Capensis bee Vol. 35 in Proceedings of the first international electronic conference on the Cape bee problem in South Africa (ed. Magnuson, P) 74–75 (PPRI, Pretoria, 1995).

[24] Neumann P, Radloff SE, Moritz RFA, Hepburn HR, Reece SL (2001). Social parasitism by honeybee workers (*Apis mellifera capensis* Escholtz): host finding and resistance of hybrid host colonies. Behavioral Ecology.

[25] Okosun OO, Yusuf AA, Crewe RM, Pirk CWW (2015). Effects of age and Reproductive Status on Tergal Gland Secretions in Queenless Honey bee Workers, *Apis mellifera scutellata* and *A. m. capensis*. J Chem Ecol.

[26] Moritz RFA, Lattorff HMG, Crewe RM (2004). Honeybee workers (*Apis mellifera capensis*) compete for producing queen-like pheromone signals. *Proceedings of the Royal Society of London*. Series B: Biological Sciences.

[27] Zheng H-Q (2010). Pheromonal predisposition to social parasitism in the honeybee *Apis mellifera capensis*. Behavioral Ecology.

[28] Sole CL, Kryger P, Hefetz A, Katzav-Gozansky T, Crewe RM (2002). Mimicry of queen Dufour’s gland secretions by workers of *Apis mellifera scutellata* and *A. m. capensis*. Naturwissenschaften.

[29] Martin SJ, Beekman M, Wossler TC, Ratnieks FL (2002). Parasitic Cape honeybee workers, *Apis mellifera capensis*, evade policing. Nature.

[30] Neumann P, Hepburn R (2002). Behavioural basis for social parasitism of Cape honeybees (*Apis mellifera capensis*). Apidologie.

[31] Hepburn HR (1992). Pheromonal and ovarial development covary in cape worker honeybees. Apis meliffera capensis. Naturwissenschaften.

[32] Moritz R, Simon U, Crewe R (2000). Pheromonal contest between honeybee workers (*Apis mellifera capensis*). Naturwissenschaften.

[33] Allsopp M (1993). Summarized overview of the Capensis problem. South African Bee Journal.

[34] Dietemann V, Pirk C, Walter Werner, Crewe R (2009). Is there a need for conservation of honeybees in Africa?. Apidologie.

[35] Baudry E (2004). Whole-Genome Scan in Thelytokous-Laying Workers of the Cape Honeybee (*Apis mellifera capensis*): Central Fusion, Reduced Recombination Rates and Centromere Mapping Using Half-Tetrad Analysis. Genetics.

[36] Keeling CI, Slessor KN, Higo HA, Winston ML (2003). New components of the honey bee (*Apis mellifera* L.) queen retinue pheromone. Proceedings of the National Academy of Sciences.

[37] Slessor KN, Winston ML, Le Conte Y (2005). Pheromone Communication in the Honeybee *(Apis mellifera* L.). J Chem Ecol.

[38] Butler CG (1961). The scent of queen honeybees (*A. mellifera* L.) that causes partial inhibition of queen rearing. Journal of Insect Physiology.

[39] Crewe RM, Velthuis HHW (1980). False queens: A consequence of mandibular gland signals in worker honeybees. Naturwissenschaften.

[40] Winston ML, Slessor KN (1998). Honey bee primer pheromones and colony organization: gaps in our knowledge. Apidologie.

[41] Hoover SER, Keeling CI, Winston ML, Slessor KN (2003). The effect of queen pheromones on worker honey bee ovary development. Naturwissenschaften.

[42] Butler CG (1959). The source of the substance produced by a queen honeybee (*Apis mellifera* L.) which inhibits development of the ovaries of the workers of her colony*. Proceedings of the Royal Entomological Society of London. Series A*. General Entomology.

[43] Melathopoulos A, Winston M, Pettis J, Pankiw T (1996). Effect of queen mandibular pheromone on initiation and maintenance of queen cells in the honey bee (*Apis mellifera* L.). The Canadian Entomologist.

[44] Pankiw T, Huang ZY, Winston ML, Robinson GE (1998). Queen mandibular gland pheromone influences worker honey bee (*Apis mellifera* L.) foraging ontogeny and juvenile hormone titers. Journal of Insect Physiology.

[45] Morgan SM, Butz Huryn VM, Downes SR, Mercer AR (1998). The effects of queenlessness on the maturation of the honey bee olfactory system. Behavioural Brain Research.

[46] Le Conte Y, Hefetz A (2008). Primer pheromones in social hymenoptera. Annual Review of Entomology.

[47] Plettner E, Slessor KN, Winston ML, Oliver JE (1996). Caste-Selective Pheromone Biosynthesis in Honeybees. Science.

[48] Yusuf, A., Pirk, C. W. & Crewe, R. Mandibular gland pheromone contents in workers and queens of *Apis mellifera adansonii*. *Apidologie*, 1–14 (2015).

[49] Plettner E (1997). Species- and Caste-Determined Mandibular Gland Signals in Honeybees *(Apis)*. J Chem Ecol.

[50] Slessor KN, Kaminski L-A, King GGS, Winston ML (1990). Semiochemicals of the honeybee queen mandibular glands. J Chem Ecol.

[51] Malka O, Karunker I, Yeheskel A, Morin S, Hefetz A (2009). The gene road to royalty – differential expression of hydroxylating genes in the mandibular glands of the honeybee. FEBS Journal.

[52] Malka O, Niño EL, Grozinger CM, Hefetz A (2014). Genomic analysis of the interactions between social environment and social communication systems in honey bees (*Apis mellifera*). Insect Biochemistry and Molecular Biology.

[53] Vander Meer, R. K., Breed, M. D., Espelie, K. E. & Winston, M. L. Pheromone communication in social insects. Vol. 162 159–344 (Westview Press, 1998).

[54] Katzav-Gozansky T (1997). Plasticity of Caste-Specific Dufour’s Gland Secretion in the Honey Bee (*Apis mellifera* L.). Naturwissenschaften.

[55] Katzav-Gozansky T, Boulay R, Soroker V, Hefetz A (2004). Queen–signal modulation of worker pheromonal composition in honeybees. *Proceedings of the Royal Society of London*. Series B: Biological Sciences.

[56] Hepburn H, Radloff S (1996). Morphometric and pheromonal analyses of *Apis mellifera* L along a transect from the Sahara to the Pyrenees. Apidologie.

[57] Wu Y (2017). Comparative transcriptome analysis on the synthesis pathway of honey bee (*Apis mellifera*) mandibular gland secretions. Scientific Reports.

[58] Vitale A, Rosso F, Barbarisi A, Labella T, D’Auria S (2010). Properties and evolution of an alcohol dehydrogenase from the Crenarchaeota *Pyrobaculum aerophilum*. Gene.

[59] Reid MF, Fewson CA (1994). Molecular Characterization of Microbial Alcohol Dehydrogenases. Critical Reviews in Microbiology.

[60] Duester G (1999). Recommended nomenclature for the vertebrate alcohol dehydrogenase gene family. Biochemical Pharmacology.

[61] Zhang Y, Xia Y, Zhu J, Li S, Dong S (2014). Putative Pathway of Sex Pheromone Biosynthesis and Degradation by Expression Patterns of Genes Identified from Female Pheromone Gland and Adult Antenna of *Sesamia inferens* (Walker). J Chem Ecol.

[62] Vogt RG (2005). Molecular basis of pheromone detection in insects. Comprehensive insect physiology, biochemistry, pharmacology and molecular biology.

[63] Lands WEM (1998). A review of alcohol clearance in humans. Alcohol.

[64] Lee FJ, Rusch DB, Stewart FJ, Mattila HR, Newton IL (2015). Saccharide breakdown and fermentation by the honey bee gut microbiome. Environmental microbiology.

[65] Chan QWT (2011). The Worker Honeybee Fat Body Proteome Is Extensively Remodeled Preceding a Major Life-History Transition. Plos One.

[66] Okosun OO, Pirk CWW, Crewe RM, Yusuf AA (2017). Glandular sources of pheromones used to control host workers (*Apis mellifera scutellata*) by socially parasitic workers of *Apis mellifera capensis*. Journal of Insect Physiology.

[67] Phiancharoen M, Pirk CWW, Radloff SE, Hepburn R (2010). Clinal nature of the frequencies of ovarioles and spermathecae in Cape worker honeybees, Apis mellifera capensis. Apidologie.

[68] Allsopp MH, Calis JNM, Boot WJ (2003). Differential feeding of worker larvae affects caste characters in the Cape honeybee. Apis mellifera capensis. Behav Ecol Sociobiol.

[69] Schäfer MO (2006). Individual versus social pathway to honeybee worker reproduction (*Apis mellifera*): pollen or jelly as protein source for oogenesis?. J Comp Physiol A.

[70] Lin H, Winston ML, Haunerland NH, Slessor KN (1999). Influence of age and population size on ovarian development, and of trophallaxis on ovarian development and vitellogenin titres of queenless worker honey bee (Hymenoptera: Apidae). The Canadian Entomologist.

[71] Mohammedi A, Paris A, Crauser D, Le Conte Y (1998). Effect of aliphatic esters on ovary development of queenless bees *(Apis mellifera* L.). Naturwissenschaften.

[72] Wossler TC, Crewe RM (1999). Honeybee queen tergal gland secretion affects ovarian development in caged workers. Apidologie.

[73] Strauss K (2008). The role of the queen mandibular gland pheromone in honeybees (*Apis mellifera*): honest signal or suppressive agent?. Behav Ecol Sociobiol.

[74] Tan K (2010). Responses of Queenright and Queenless Workers of *Apis Cerana* to 9-keto-2(E)-decenoic Acid, a Pheromonal Constituent of the Mandibular Gland. J Chem Ecol.

[75] Moritz RFA, Hillesheim E (1985). Inheritance of dominance in honeybees (*Apis mellifera capensis* Esch.). Behav Ecol Sociobiol.

[76] Traynor KS, Le Conte Y, Page RE (2014). Queen and young larval pheromones impact nursing and reproductive physiology of honey bee (*Apis mellifera*) workers. Behav Ecol Sociobiol.

[77] Démares FJ, Yusuf AA, Nicolson SW, Pirk CWW (2017). Effect of Brood Pheromone on Survival and Nutrient Intake of African Honey Bees (*Apis mellifera scutellata*) under Controlled Conditions. J Chem Ecol.

[78] Neumann P, Moritz R (2002). The Cape honeybee phenomenon: the sympatric evolution of a social parasite in real time?. Behav Ecol Sociobiol.

[79] Reece SL (2002). A scientific note on the ovarial and pheromonal development of drifted and non-drifted Cape honeybee workers (*Apis mellifera capensis*). Apidologie.

[80] Genç M, Aslan A (1999). Determination of trans-10-hydroxy-2-decenoic acid content in pure royal jelly and royal jelly products by column liquid chromatography. Journal of Chromatography A.

[81] Robertson HM (1999). Diversity of odourant binding proteins revealed by an expressed sequence tag project on male *Manduca sexta* moth antennae. Insect Molecular Biology.

[82] Crewe, R. Compositional variability: the key to the social signals produced by honeybee mandibular glands in The biology of social insects (eds Michener, C. D. & Evan, H. E.) 318–322 (Westview Press, 1982).

[83] Pettis JS, Higo HA, Pankiw T, Winston ML (1997). Queen rearing suppression in the honey bee - evidence for a fecundity signal. Insect. Soc..

[84] Williams GR (2013). Standard methods for maintaining adult *Apis mellifera* in cages under *in vitro* laboratory conditions. Journal of Apicultural Research.

[85] Carreck NL (2013). Standard methods for *Apis mellifera* anatomy and dissection. Journal of Apicultural Research.

[86] Hess, G. Über den Einfluß der Weisellosigkeit und des Fruchtbarkeitsvitamins E auf die Ovarien der Bienenarbeiterin, Diss. Naturwiss. ETH Zürich, Nr. 1200, 0000. Ref.: Schneider-Orelli, O.; Korref.: Seiler, J. (1942).

[87] Velthuis HHW (1970). Ovarian Development in *Apis mellifera* Worker Bees. Entomologia Experimentalis et Applicata.

[88] Simon UE, Moritz RFA, Crewe RM (2001). The ontogenetic pattern of mandibular gland components in queenless worker bees (*Apis mellifera capensis* Esch.). Journal of Insect Physiology.

[89] Pirk CW (2013). Statistical guidelines for *Apis mellifera* research. Journal of Apicultural Research.

[90] Livak KJ, Schmittgen TD (2001). Analysis of Relative Gene Expression Data Using Real-Time Quantitative PCR and the 2^−ΔΔCT^ Method. Methods.

[91] R: A language and environment for statistical computing (Vienna, Austria., 2015).

